# Editorial: Quantitative Biology: Dynamics of Living Systems

**DOI:** 10.3389/fphys.2016.00196

**Published:** 2016-06-02

**Authors:** Noriko Hiroi, Viji M. Draviam, Akira Funahashi

**Affiliations:** ^1^Systems Biology Laboratory, Department of Biosciences and Informatics, Keio UniversityYokohama, Japan; ^2^Cell and Molecular Biology, School of Biological and Chemical Sciences, Queen Mary University of LondonLondon, UK

**Keywords:** quantitative biology, bioimaging, parameter optimization, generic technologies, cell division

With the emergence of Systems Biology, there is a greater realization that the whole behavior of a living system may not be simply described as the sum of its elements. To represent a living system using mathematical principles, practical quantities with units are required. Quantities are not only the bridge between mathematical description and biological observations; they often stand as essential elements similar to genome information in genetics. This important realization has greatly rejuvenated research in the area of Quantitative Biology.

Because of the increased need for precise quantification, a new era of technological development has opened. For example, spatio-temporal high-resolution imaging enables us to track single molecule behavior *in vivo*. Clever artificial control of experimental conditions and molecular structures has expanded the variety of quantities that can be directly measured. In addition, improved computational power and novel algorithms for analyzing theoretical models have made it possible to investigate complex biological phenomena.

This research topic is organized on two aspects of technological advances which are the backbone of Quantitative Biology: (i) visualization of biomolecules, their dynamics and function, and (ii) generic technologies of model optimization and numeric integration. We have also included articles highlighting the need for new quantitative approaches to solve some of the long-standing cell biology questions.

In the first section on visualizing biomolecules, four cutting-edge techniques are presented. Ichimura et al. provide a review of quantum dots including their basic characteristics and their applications (for example, single particle tracking). Horisawa discusses a quick and stable labeling technique using click chemistry with distinct advantages compared to fluorescent protein tags. The relatively small physical size, stability of covalent bond and simple metabolic labeling procedures in living cells provides this type of technology a potential to allow long-term imaging with least interference to protein function. Obien et al. review strategies to control microelectrodes for detecting neuronal activity and discuss techniques for higher resolution and quality of recordings using monolithic integration with on-chip circuitry. Finally, the original research article by Amariei et al. describes the oscillatory behavior of metabolites in bacteria. They describe a new method to visualize the periodic dynamics of metabolites in large scale cultures populations. These four articles contribute to the development of quantitative methods visualizing diverse targets: proteins, electrical signals and metabolites.

In the second section of the topic, we have included articles on the development of computational tools to fully harness the potential of quantitative measurements through either calculation based on specific model or validation of the model itself. Kimura et al. introduce optimization procedures to search for parameters in a quantitative model that can reproduce experimental data. They present four examples: transcriptional regulation, bacterial chemotaxis, morphogenesis of tissues and organs, and cell cycle regulation. The original research article by Sumiyoshi et al. presents a general methodology to accelerate stochastic simulation efforts. They introduce a method to achieve 130 times faster computation of stochastic models by applying GPGPU. The strength of such accelerated numerical calculation are sometimes underestimated in biology; faster simulation enables multiple runs and in turn improved accuracy of numerical calculation which may change the final conclusion of modeling study. This also highlights the need to carefully assess simulation results and estimations using computational tools.

The final section of our research topic illustrates open questions in our understanding of dynamic cellular events— molecular crowding and cell division—that could benefit using quantitative biology approaches. The review by Aon and Cortassa focuses on macromolecular crowding in a cell. The authors discuss that the self-organizing capability of the cytoskeleton can orchestrate metabolic flux, while the fractal organization can frame the scaling activity. The review aims to shed light on ways to integrate the structural and functional linkage *via* crowding. Molecular crowding of each organelle may be affected by the flow into and out of the compartment. Vincent et al. focus on proteins in endoplasmic reticulum which have to enter through membrane-embedded translocons. They present concrete estimates on the flow of proteins entering the ER lumen. Berry and Soula present original findings on the importance of transient subdiffusion for protein distribution in space, when transient subdiffusion is restricted to a subregion of the space. Their simulations reveal a strong accumulation at equilibrium in the subdiffusion region that is controlled by the long-time asymptotic Brownian regime rather than the initial short-time subdiffusion.

Cell division is a fundamental process that goes awry in cancers yet there has been a puzzling absence of prominent oncogenic mutations in key cell division regulators. By measuring cell size and duration of cell cycle in early embryonic development of *C. elegans*, Arata et al., reveal a power law relationship between cell cycle duration and cell volume. They propose that geometric constraints between intracellular structures may coordinate cell cycle with the size of the original cell. In the opinion article Chin et al., highlight the need to build multiscale models for understanding pathways that jointly control the plane of cell division. Using structural knowledge of multi-protein complexes, Lee and Bolanos-Gracia review the dynamics of checkpoint signal amplification during cell division which ensures the accurate segregation of chromosomes. Mathematical models of dynamic cytoskeletal processes may be required to understand and intervene with tumor cell behavior.

In summary, our topic gives a flavor of new candidate probes for rigorous quantification, which needs to be perpetually emphasized in Quantitative Biology. Non-linear dynamic behavior of living systems is likely to be a leading challenge that needs to be described quantitatively. We hope the articles in this Research Topic will help you find your own, attractive perspective of biology via quantitative analyses.

## Author contributions

NH prepared the first draft, and VD and AF made critical revision of the manuscript.

## Funding

The International workshop on Quantitative Biology 2012 to 2014 were supported by The Royal society, UK, Industrial Institute of the University of Tokyo, Japan, Keio University, Japan, Transdisciplinary Research Integration Center of Research Organization of Information and Systems, RIKEN CDB, Japan, VLSCI Life Science Computation, Australia, and DaiNippon Sumitomo Pharma, Japan.

### Conflict of interest statement

The authors declare that the research was conducted in the absence of any commercial or financial relationships that could be construed as a potential conflict of interest.

